# Playing with data differently: engaging with autism and gender through participatory arts/music and a performative framework for analysis

**DOI:** 10.3389/fpsyg.2024.1324036

**Published:** 2024-06-17

**Authors:** Nicola Shaughnessy, Ruth Herbert, Emma Williams, Jackie Walduck, Rocio von Jungenfeld, Hannah Newman

**Affiliations:** ^1^School of Arts, University of Kent, Canterbury, United Kingdom; ^2^Department of Music & Audio Technology, School of Arts, University of Kent, Medway, United Kingdom; ^3^School of Psychological Sciences, University of Surrey, Guildford, United Kingdom; ^4^Department of Academic Studies, Royal Academy of Music, London, United Kingdom; ^5^School of Engineering, University of Kent, Canterbury, United Kingdom

**Keywords:** autism, gender, music, participatory arts, performative, play, transdisciplinary

## Abstract

There are increasing demands for Participatory Arts-Based (PAB) programs involved in health research to better evidence outcomes using robust quantitative evaluation methodologies taken from science, such as standardized questionnaires, to inform commissioning and scale-up decisions. However, for PAB researchers trying to do this, barriers arise from fundamental interdisciplinary differences in values and contexts. Researchers are required to navigate the tensions between the practice-based evidence produced by the arts and the evidence-based practice sought by psychologists. Consequently, there is a need for interdisciplinary arts-science collaborations to produce alternative methods of evaluation that are better aligned to PAB approaches, and which combine systematic rigor with a sensitivity to the values, contexts and strengths of this approach. The current article centers on the development of an alternative transdisciplinary analytic tool, the Participatory arts Play Framework (PP-Framework), undertaken as part of an arts-psychology collaboration for a UK AHRC-funded PAB research project: Playing A/Part: Investigating the identities and experiences of autistic girls. We present details of three stages in the development of the PP-Framework: 1. preliminary emergence of the framework from initial video analysis of observational data from participatory music and sound workshops run for 6 adolescent autistic girls (aged 11–16); 2. identification and application of modes of engagement; and 3. further testing of the framework as an evaluation tool for use in a real-world setting, involving professional musicians engaged in delivery of a creative music project at a center for homeless people. The PP-Framework maps types of participation in terms of performative behaviors and qualities of experience, understood as modes of play. It functions as a vehicle for analyzing participant engagement, providing a tool predicated on the processes of working in creative participatory contexts while also being sensitive to the esthetic qualities of what is produced and capable of capturing beneficial changes in engagement. It offers a conceptual approach for researchers to undertake observation of participatory arts practices, taking account of embodied engagement and interaction processes. It is informed by understandings of autistic performativity and masking in conjunction with an ecological understanding of sense making as being shaped by environments, social relations and sensing subjectivity. The framework has the potential to be a bi-directional tool, with application for both practitioners and participants.

## 1 Introduction

### 1.1 Research challenges in arts-science and participatory arts: evidence-based practice vs. practice-based evidence

There have been increasing demands in recent years for participatory arts-based (PAB) programs involved in health research to better evidence outcomes through the use of robust quantitative evaluation methodologies so as to more effectively inform commissioning and scale-up decisions (Bungay and Vella-Burrows, 2013; Crossick and Kaszynska, 2016; Zarobe and Bungay, 2017; Daykin et al., 2017b; Clift et al., 2021). PAB programs refer to a diverse range of practice-based creative community activities involving active engagement (e.g., producing, performing) and non-hierarchical collaboration between facilitators and participants which are considered helpful to aspects of mental health and well-being. They can involve the use of one or more art forms, including music, dance, theater, visual arts, storytelling, poetry, and film. PAB programs are usually facilitated by artists or arts practitioners in the form of group workshops (O’Donnell et al., 2022) and delivered in community settings, such as schools, youth centers and justice sites. This contrasts with arts therapies and interventions which are delivered by trained therapists in clinical settings, using clinical approaches alongside art and creativity to support emotional work (Kalmanowitz et al., 2019). PAB programs are also distinct from other community-led arts programs, such as choral singing or life drawing, which often emphasize the use of artistic engagement as a resource for building social inclusion and supporting mental wellbeing, but are delivered *to* receptive participants, rather than being centered on participatory involvement *in* creative processes, such as through composing, co-creation or design.

Recent critiques of PAB programs call for, among other things, more robust, systematic and transparent methods of evaluating health and social impacts. Standardized mental health measures enjoy high status in medico-scientific fields, yet only a relatively small proportion of PAB studies have employed these to evaluate outcomes (Daykin et al., 2017b). PAB researchers have reported several barriers to their use, including difficulties in participants’ understanding of the included questions (Efstathopoulou and Bungay, 2021), problems in implementation (Wood et al., 2013), and issues with the attitudes of participants toward completing the measures in PAB contexts (Daykin et al., 2017a). Daykin et al. (2017a), who initially planned a mixed methods study, abandoned the quantitative data on the basis that the participating young people in the justice settings were observed to engage ‘in banter, conferring and joking’ (p. 944) with each other during completion of a questionnaire, raising doubts about the validity of evidence collected via this means. Several arts practitioners and researchers have voiced concerns around the jarring of values and the meaning of terms such as ‘effective’ and ‘outcome’ which may be differently inflected for creative and clinical researchers (Heinemeyer, 2017). Others have questioned why it is that the outcomes of Randomized Control Trials and quantitative assessments are seen as the only viable form of evidence, when there are valuable qualitative insights from PAB participants (Crossick and Kaszynska, 2016; Clift et al., 2021). There are calls for interdisciplinary approaches to evaluating PAB that embrace different kinds of evidence (Reason and Rowe, 2017), encompassing esthetics (artistic content, quality and affective experience) and ethics (benefits for participants). Related concerns are associated with what constitutes data and how this might be reconceptualized as a dynamic process, rather than a fixed body of material to be collected and analyzed. As participatory arts and practice research are iterative approaches that value process as well as artistic products combining ethical and esthetic motivations, new models of data are needed appropriate to this fluidity. This would offer a potential third space for transdisciplinary enquiry (Hansen, 2017).

### 1.2 Differences in values and contexts between participatory arts and science disciplines

While being cautious of the crudity of the “two cultures” binaries (Shaughnessy and Barnard, 2019) and associated oppositions (hard science v soft arts), participatory arts and science disciplines (particularly health/medicine) differ markedly in terms of their values and the contexts in which methods and evaluations are undertaken. This makes the simple “transplantation” of a method of evaluation from one discipline into another problematic.

*Values and purpose:* One key factor that differentiates between values in arts (particularly the emerging field of ‘creative health’) and those in health science is the purpose of research activity. In an essay on ‘Valuing Performance’, Shaughnessy (2016) observed that PAB research methodologies explore research questions *through* artistic practice. A question-based approach generates open and dialogic structures appropriate to participatory practices and the impetus to work “with” rather than “applying to” participants. This conception of working “with” connects to the ideologies of participatory community research in autism studies (see 1.4 below) and critiques of the deficit-focused medical model in preference to a conceptualization of ‘difference’ informed by the neurodiversity movement and a social model of disability. In contrast to social scientists who typically measure efficacy in terms of quantifiable improvements in well-being, symptoms or behavior, PAB researchers prioritize enhancements in agency (via artistic expression), community engagement (via creative collaboration) and esthetics (qualities of the art produced) in evaluative reports (Williams et al., 2023). Consequently, the standardized questionnaires commonly used in health science are not designed to capture the kinds of change valued by arts researchers.

*Contexts:* The aim to work “with” participants also means that the contexts in PAB studies are fundamentally relational. The processes involved are informed by an epistemological perspective of knowledge production as co-created through interactive, embodied and experiential modes of sense-making. The dialogic nature of engagement enables artists and practitioners to draw on the experiences of people taking part, ensuring participation is meaningful for them (Lowe, 2012) while also making it possible to tailor engagement and therapeutic benefits for mental health to specific individuals and groups (Pavarini et al., 2021). Both the art and the artist are transformed through their interactions, as well as being the catalyst for change themselves. A recent systematic review (Williams et al., 2023) of participatory art-based studies which evaluated outcomes related to mental health and wellbeing in young people found clear similarities between arts-based processes and clinically therapeutic processes, such as those involved in Person-Centered approaches to Counseling (PCC; Rogers, 1957, 1959). In both the therapeutic work appeared to rest on establishing relationships of trust between practitioners and participating youth as well as between the children and young people themselves. PAB practitioners aimed to create a non-judgmental, accepting, ‘protected’ space in which the children and young people felt able to take social and emotional ‘risks’ through engagement with arts stimuli and practices to help them reconnect to their thoughts, feelings and bodies. Establishing a ‘protected’ social space based on trust and acceptance facilitated deeper and more sustained engagement which, in turn, opened pathways to the development of a more positive or ‘authentic’ self-narrative. It also supported young people to express themselves and create their own meanings via embodied engagement with the art activities, restoring or building a sense of agency and self-direction.

During our ‘Playing A/Part’ project that is the focus of discussion for this paper, the use of standardized questionnaires in a context that is relational and centered on co-creation gave rise to significant tensions between the psychology and arts team. At times, their use even threatened to de-rail relationships between practitioners and young people that had been built on trust and accepting participants on their own terms. In contrast to the spirit of co-creation, the questionnaires were viewed by some participants as being an imposition. They ran counter to the need for establishing non-judgmental relationships, in the sense that some autistic girls reported feeling objectified and that they were being viewed through the same ‘deficit’ lens they associated with their experiences of medical intervention. For others, the questionnaires generated an anxiety comparable to that felt during tests at school, as they feared not being able to complete them in the ‘correct’ way. In common with Daykin et al. (2017a), we experienced situations in which the autistic girls taking part in our project appeared not to take completion of the standardized mental health measures seriously, engaging in banter with each other during completion, possibly as a form of resistance to their imposition. Such reactions raised ethical issues for the research team. While all aspects of the study had been clearly explained to the young people to ensure informed consent/assent, we became concerned that some may have been to some extent coerced into completing the measures in order to take part in the arts-activities which they wanted to do, or that in giving consent/assent they had focused on consenting to the arts-activities rather than other aspects of the study. Such findings support calls for a reconceptualization of processes of consent/assent toward a ‘… relationally constituted process, more aligned with the overall epistemological frameworks of participatory research’ (Van Goidsenhoven and De Schauwer, 2022, p. 1323) whereby consent and assent are continuous processes that go beyond a signed form, conducted in dialogic contexts between researchers, people with lived experience, carers, families, advocates and using methods that enable inclusion and agency.

### 1.3 Strengths of practice-based approaches

The challenges faced by participatory arts-based researchers have some resonance with those encountered by qualitative researchers a couple of decades ago, who were experiencing difficulties in getting their research published in science journals (Elliott et al., 1999; Yardley, 2000). In the latter case, evaluative criteria designed to assess the quality of work in one research context (quantitative) were being applied to judge an approach with very different philosophical underpinnings, principles and methods (qualitative). Likewise, PAB research has tended to be critiqued through the lens of science for what is not there rather than for what is. PAB studies have been advised to adopt methods of evaluation used in the health science research context (such as standardized questionnaires) to increase their utility and acceptance. Consequently, their potential to offer rich, contextualized and robust evidence relating to processes of change arising from practice and their value as a research tool designed to increase our understanding of the lived experience of participants are in danger of being overlooked. Studies reporting on PAB programs using qualitative approaches often provide detailed process-focused analyses of interview, focus group and observational data. Artists and arts practitioners are able to offer nuanced and reflexive accounts of their practice that recognize the complexities of their own and young people’s involvement, the cultural context in which the arts activities take place and the artistic process (Clift et al., 2021). They can also report on aspects of the arts practice and process that facilitate or hinder change, and the contribution of the role played by the art form itself to changed understanding. PAB approaches can be used as a powerful research tool to help researchers discover more about the subjective lived experiences of the people they are working with. Increased understanding of their phenomenology gained from such evidence can bring about change indirectly through helping us better tailor existing structures of education, support and care to the needs and sensitivities of different groups. In our project the psychologists on the research team incorporated PAB methods into interviews to strengthen their capability of these to elicit rich information about how autistic girls made sense of themselves and their experiences.

### 1.4 Participatory community research processes: principles, practices and variables

The title for our project, ‘Playing A/Part: investigating the identities and experiences of autistic girls through drama, interactive media and participatory arts’ gestures toward its commitment to participatory community research. ‘Playing A/Part’ refers to a range of features and experiences associated with autistic girls and women (e.g., sociality, masking, performativity), as well as indicating the role played by the autistic community as part of the project. It was conceived during a pilot project with autistic and female identifying students at the University of Kent (2016). The research design developed from a program of co-produced practical workshops in which community members experimented with narrative techniques (e.g., writing, improvisation, photography, film). This led to a growing network of autistic researchers, artists and educators, advising on the development of the proposal and its terminologies (e.g., use of identity first language and attention to marginalized genders).

The participatory approach responds to concerns that ‘the degree of community involvement in UK autism research remains close to the bottom of the ladder’ and that only 5% of research is reported to have been dedicated to support and education (Pellicano et al., 2014). Participatory democratizing approaches are having a significant impact across disciplines, challenging traditional methods and power structures through a commitment to working *with* people with lived experience rather than research being done *to, on* or *for* individuals. In the autism field, the slogan “nothing about us without us” has been adopted to spearhead the campaign for research that is inclusive of lived experience and relevant to the needs and realities of autistic people. However, what this means in practice can vary from research that involves consultation with autistic advisory groups (regarding design, recruitment and findings) to a fully holistic approach with autistic people engaged in all aspects of the project: design, data collection and analysis and dissemination. A fully participatory protocol would involve engaging autistic participants as researchers in the design, analysis and authorship of the project in addition to having lay community members as advisors. It would also necessitate adequate remuneration for contributions (Pellicano et al., 2022). In the ‘Playing A/Part’ project autistic lived experience formed a body of knowledge with equal importance as the disciplines of art and science in a triangulation of expertise fundamental to the interdisciplinary framework. The project involved a Steering Group of autistic women as well as having autistic representation as part of the project’s Advisory Board. It also included autistic researchers, creative practitioners delivering workshops, an autistic film maker and an inclusive publication strategy.

### 1.5 Conceptual background to project

Conceptually, the approaches for the Playing A/Part project were informed by cognitive neuroscience and theories of sense making and agency through 4E cognition whereby sensorimotor and semantic understanding are linked with body/action and language, through the physical (embodied), sensory interactions with environmental affordances and stimuli (embedded), connections beyond the individual to the social and interpersonal (extended), and the links between action and perception (enactive). This involves an integration of first and third person perspectives in conjunction with an emphasis on the role of social interactions and environmental factors in understanding identity formation and sense of being in the world. Cognitive science continues to address the challenges of historical mind/body dualisms through the embodied approach and the 4E framework whereby there is no ontological division between the individual and the environment and the embodied mind is understood as dynamic and emergent (Varela et al., 1991; Kyselo, 2016). The conceptual framework of 4E cognition has important implications for understanding autism through its emphasis on lived experience, social and material environments and the affective and perceptual dimensions shaping neurodivergent consciousness. As one of the authors (Herbert) has noted elsewhere, subjective awareness can be understood as constituting “the gestalt sum of a network of cognitive, perceptual, emotional and physiological interactions” (2011, p. 31). In terms of music, moreover, the ecological approach (integrating theoretical perspectives from 4E cognition), similarly conceptualizes musical identities as ‘fluid and constructed through embodied and situated action (MacDonald and Saarikallio, 2022, p. 9).

### 1.6 Rationale for current paper

We argue that for interdisciplinary arts-science collaborations to realize their full potential in more powerful and integrated ways, a deeper understanding of their fundamentally different values and contexts is required from the outset, as well as a greater appreciation of the strengths that PAB approaches can offer the partnership. There is an urgent need for interdisciplinary arts-science collaborations to produce alternative transdisciplinary methods of evaluation that are better aligned with PAB approaches, combining systematic rigor with a sensitivity to the values, contexts and strengths of that approach, as well as to diversity and inclusion. In line with this, the current article presents the development of an alternative observational analytic tool, the Participatory Arts Play (PP) Framework, which involved engaging differently with data. Within this approach data is reconfigured as being ‘made’ (rather than found), assembled (rather than collected as a fixed entity), in recognition that researchers and those they are working with ‘bring data into being’ through dialogic and relational processes (Gale, 2018; Ellingson and Sotirin, 2020). The need for an observational tool with sensitivities to different modes of engagement in participatory arts activities emerged during our initial discussions of how to engage in the process of ‘making data’ as an interdisciplinary team (Ellingson and Sotirin, 2020). We were already situated in a creative and conceptual space between disciplines in terms of how participatory processes are evaluated (i.e., the purpose and values of the research activities). For social scientists, the efficacy of creative practices are evidenced through measures of benefits for participants, in terms of symptom improvement (particularly mental health) and change, while arts-based scholarship identifies development and change in terms of esthetic (creative content) and ethical (purposeful) aspects (e.g., community engagement/ through collaboration; Williams et al., 2023).

## 2 Development of the framework through participatory music and sound workshops study

### 2.1 Introduction

Playing A/Part was an interdisciplinary collaboration between arts-based researchers at the University of Kent (Drama, Music and Digital Arts), psychology researchers at the University of Surrey and a steering group of autistic women (inclusive of marginalized genders). The project used participatory arts methodologies in conjunction with a mixed methods psychological framework, informed by an ecological approach (Bennett, 2015; Harpin and Nicholson, 2016). A mixed-methods approach to evaluation and analysis of the workshops allowed for the triangulation of multiple data sources to understand change and the integration of first- and third-person perspectives (Fonagy, 2009).

The focus of this paper is a music workshop program in the context of the larger project. This emerged in the context of the Covid 19 pandemic which disrupted data collection and delayed the project timetable. A program of online and hybrid creative projects formed the basis for the project’s trial in schools. However, the music workshops occurred in a post lockdown period when there was an opportunity for in person activities to resume. Built into the project design was a longitudinal commitment to sustain and embed the creative practices, responding to concerns about the potential for helicoptering in and out of community contexts, articulated in relation to both participatory arts and participatory community research (Fautley, 2014).

Although the music workshop program was developed in a participatory context, we define it as being committed to the *principles* rather than the practice of participatory research (the model for the main project) due to its positioning in the context of the pandemic and toward the end of the project’s extended time frame. The workshops were delivered in collaboration with the project’s partner school, Limpsfield Grange (the only specialist school for autistic girls in the United Kingdom) who had requested us to work with their boarding community as part of an extended day extra-curricular program. The original program for the main project trial had been developed with this group of learners, testing and adapting the creative practices devised with the autistic university students in the pilot project so the music workshops followed the same principles (also working with some of the same learners). The program was optional and ran after school which meant the girls contributing were choosing to do so (alternative concurrent activities were offered by the school). Due to Covid restrictions, there were limits on the number of practitioners leading the workshops (two) and a testing regime was in place as part of the health and safety framework. Consequently, we could not involve the full team in workshop delivery and consultations with the Steering Group were limited to discussion with the Chair and specific individuals due to resources and availability. Although, there was autistic representation in the coding and analysis, the desired citizen science approach would have involved more autistic contributors, and this would certainly be part of the protocol for the next stage of research.

### 2.2 Study aims

As the workshop program for the music project was positioned toward the end of an iterative research process, we conceived it as an experimental sub-project within the larger research program and as an opportunity to play with what we considered to be transdisciplinary approaches in the ‘third space’ of arts/science interaction. The workshops provided an informal and relational space for exploring perceptions and questions about autistic girls’ lived experience and agency. Specific aims were: (a) To use music and sound as tools to explore creativities and the lived experience of autistic girls (e.g., approach to music/sound-making, perceptions of self, sensory awareness); (b) To understand ways in which music and sound are used to negotiate everyday life (e.g., as a means of self-regulation to modulate mood, modulate subjective experience, manage sensory-affective relationship to external environment, to frame routines); (c) To develop musical skills, applying the girls’ musicality as expressed through their music and sound preferences; (d) To support participants’ well-being through creative empowerment, developing a sense of artistic To encourage group work and build community between participants.

### 2.3 Music and sound workshops design

Individuals participated in an 8-week workshop series (titled ‘Creative Club’) led by two experienced arts practitioners (an applied music specialist and a physical theater/movement expert). The project was designed to encourage creative expression within groups and as individuals, to offer a range of routes into music creation, drawing on participant experience and appreciation of available sounds. Workshops explored ways to generate and record sonic/musical materials, using instruments and “found sounds”. Activities included: “Being a band”—playing rhythmically in a group, improvising collectively; composing short musical signatures; recording found sounds from the school environment (relating to preferred everyday sounds); creating foley sounds (reproductions of everyday sound effects) in response to an image; experimenting with audio effects such as delay, phasing, filtering; multisensory den-building; individual compositions, compiling and manipulating selected sounds. Each composition was installed on a small speaker and positioned inside the multisensory dens.

A significant number of activities possessed multimodal components, intended to facilitate engagement and immersion (for example, the recording of found sounds in the school grounds). A multimodal emphasis was selected as being appropriate to yielding understanding of how autistic participants experience themselves, others and the world around them, in keeping with theoretical understanding of embodied cognition and intersubjectivity. Workshops were planned to become increasingly multimodal as they progressed, culminating in installations created at the end of the project in the form of multisensory dens, intended to afford researcher insight into participants’ subjective sense of self and relationship with their immediate environments. These were conceived as “safe spaces” (Shaughnessy, 2022) over which the girls had authorial control as multimedia installation designers, with sound design installed in the dens as a key project outcome.

Workshops occurred weekly, after the end of the school day and were 2 h in length. Prior to the commencement of the Creative Club, a ‘social story’ was shared with the school, giving an overview of the series, also introducing practitioners and the research team. In the first workshop, students and practitioners co-produced a code of practice for the series. This included the right to pass on activities, to take time out, the availability of a chill-out space and agreed approaches to taking part in group activities (e.g., listening to others, turn taking). Each session began with a physical activity/musical warm-up to transition from the school context into a creative space and an overview of the session structure was displayed on a flip chart.

### 2.4 Participants

Six girls with a diagnosis of autism, aged 11–16, attending a specialist school setting for autistic girls were recruited. Participants self-selected to become part of the study (volunteer sampling) in response to pre-scripted verbal summary which teachers shared with students. All students who volunteered declared an involvement or interest in music. Five out of six regularly listened to music, three had some experience of playing musical instruments (through instrumental lessons at primary school and informal experimentation/self-teaching). None were currently receiving formal musical instrument training and music was not a subject accommodated within the school curriculum. Prior to commencement of the workshop series potential participants received project information sheets (written using language suited to their age and developmental level), enabling them to give informed assent. These were accompanied by information sheets for parent(s)/guardians and consent forms, which were signed by parent(s)/guardians and returned to school. In this paper all participants are referred to using pseudonyms to preserve anonymity. Ethical approval was granted by the University of Kent’s Central Research Ethics Committee.

### 2.5 Procedure and materials

Video data constituted the primary source informing the development of the PP-Framework. During the project, each workshop session was video-recorded, generating a large amount of video data (c. 14 h). Static cameras captured group workshop activities, while hand-held cameras were used by participants to document individual experiential perspectives (particularly with relation to den-making). Representation of participant and practitioner perspectives was intrinsic to data collection. In tandem with video footage, journals were used by participants to record creative ideas during workshop sessions and to reflect on activities at the end of each session. Before and after sessions participants also completed what we called ‘vibe checks’ which formed the participant section of a specially designed Participatory Arts Outcome Measure (PArts/OM). This part of the measure was designed to assess perceived changes in participants’ present-centered experience of the sessions, using emoticons (e.g., happy, sad, grumpy faces). Practitioners kept observational notes and also monitored participant engagement using the practitioner section of the PArts/OM. This section of the measure aimed to capture changes perceived by the practitioners in self-confidence, self-expression, agency, peer social engagement, and/or creativity in autistic girls/adolescents that have been brought about by their taking part in participatory arts’ workshops, as captured via a 7-point Likert scale, where 1 = strongly disagree and 7 = strongly agree.

To understand the broader context in which the PP-Framework was developed, it will be useful to briefly detail other instruments used to collect data around the workshops. These included qualitative instruments, (e.g., semi-structured interviews) and quantitative standardized baseline measures [Social Self Efficacy Scale (SSES); Creative Self-Efficacy (CSE); Warwick-Deinburgh Mental Wellbeing Scale (WEMWBS) and the Healthy and Unhealthy Music Scale (HUMS); Saarikallio et al., 2015]. As the principal focus of this paper is the development of the PP-Framework, findings from these data methods are not considered here.

### 2.6 Data analysis

There is a lack of consensus regarding practical and theoretical guidelines for the process of video analysis (Ramey et al., 2016). Video is ‘not a “neutral” source of data; inherent in the collection of video is the perspective of the camera and the researcher’s decisions about which perspective to foreground in the data’ (Ramey et al., 2016, p. 1038). One strategy, utilized by a number of researchers, is to adopt an overarching theoretical framework (e.g., Actor Network Theory) as the basis for the development of a method appropriate for a specific project. For example, Lee and McFerran (2015) developed a distinct video microanalysis method for use in a music therapy context. The approach to video analysis in the current project was necessarily multimodal, incorporating visual and sonic data characteristics (Bezemer and Mavers, 2011), shaped by emerging themes discovered in the data and also by the aims of the music and sound project, particularly its focus on exploring the lived experience of autistic girls, their creative approaches to music/sound-making and the roles they adopted in group work. This approach, and its emphasis on process and interactions (intra-actions) between people, instruments, musiking and the school environment can be aligned with Ken Gale’s conceptualization of ‘data events’ (Gale, 2018, p. 331) moving beyond conceptualizations of data as fixed objects to a more dynamic understanding of data as appropriate to practice based research:

Data as event/full is about transmutations and flux, where multiple entanglements of materiality and discourse are the vibrant matter of agentic assemblages. These kinds of entanglements intra-act with the inquiry itself (Van Goidsenhoven and De Schauwer, 2020).

In assembling data, we were seeking to respect and to reconcile disciplinary differences alongside the integrity of the participant contributions. Video documentation from two static cameras was viewed alongside field notes. Hence two researchers who had worked within the workshops as creative facilitators collaborated with three researchers outside of the practical activities to analyze the video documentation. This meant we were accessing the process from multiple perspectives using the documentation from the cameras and the academic and community knowledge we brought to the work. This assemblage of data was acknowledged as partial with interactions happening outside of the cameras and aspects of the environment and group dynamics contributing to the process in ways that could not be documented or evidenced. Nevertheless, the data assemblage offered the potential for insights that addressed our research questions in terms of autistic identities, neurodivergent creativity and meaning making.

Initially research team members viewed video content independently to become familiar with it. Given the lengthy duration of the footage, researchers then focused on visual data characteristics (behaviors, settings, motions, gestures, group interaction) and sonic data characteristics (speech, sound) with relation to two participants each. Discussion and cross comparison of written observations, contextualized by the aims of the study, was used to determine the nature of the unit(s) of analysis. A primarily thematic unit of analysis was adopted (Fazeli et al., 2023) centered on ‘significant’ or ‘difficult’ moments, events and points of change, within and across the workshops (Trowsdale and Hayhow, 2013; Shaughnessy, 2022). ‘Significant moments’ is a term used to denote a shift or change in perspective or understanding for participants or practitioners engaged in creative research processes (Trowsdale and Hayhow, 2013; Shaughnessy, 2022). Such moments can also be uncomfortable or unsettling but it is in these defamiliarizing spaces that learning occurs. Significant or difficult episodes could relate to individual and group agency, engagement and interaction. Video analysis led to the development of a novel interpretative framework, provisionally titled the Participatory Arts Play Framework. The Participatory Arts Play Framework (henceforth PP-Framework) thus forms the primary organizing focus of the findings section.^1^

## 3 Findings

### 3.1 Initial findings and analytical process leading to the development of the framework

The PP-Framework emerged from the initial analysis of video data by researchers representing the multi-modalities of participatory arts: applied music, drama and movement with autism specialists (including researchers and practitioners with lived experience). The challenges of representing and recording our observations of engagement in creative participatory practices were evident in the earliest stages of working with the video documentation. The arts-based practitioners used close observation and thick description focused on how the participants engaged with the process of music making and the qualities of what they produced, individually and in groups. Discussion of a coding framework addressed the need for different researcher perspectives to ensure reliability as well as the richness and nuances of the behaviors being documented. There was, however, a large amount of data and a system was needed to rationalize the selection of material for analysis. One approach to look at the same time stamps across the sessions was rejected because of concerns about what might be missed. Hence the decision to identify “significant moments” as the basis for close analysis. This video analysis as well as field note documentation identified a series of performative behaviors as modes of ‘playing’ (the basis for engagement in participatory arts) and this led to the emergence of the PP-Framework as a finding. This draws on an empirical process of observing and mapping musical behavior which was first developed by Ockelford et al. (2005) in the “Sounds of Intent” (SoI) framework (Ockelford et al., 2005, 2011; Welch et al., 2009). SoI originated within the context of special needs music education and was intended to map musical development in children with learning difficulties and was later extended to map other forms of creative sensory engagement such as movement & dance (Ockelford, 2013, 2015, 2020). The SoI framework identifies six levels of musical development which have a triad of domains at each level. The three behavioral ‘domains’ are defined as: Reactive (R responses to sound); Proactive (P creating sounds and music independently); Interactive (I interacting with others through music and sound).

The six levels progress from Level 1: ‘Confusion and chaos’, with limited awareness of sound structures’; Level 2; ‘Awareness and intentionality’, an emerging awareness of sound and its possibilities; Level 3: ‘Relationships, repetition and regularity’ whereby there is awareness of the significance between sonic events; Level 4: ‘Sounds forming clusters’, perception of groups of sounds, and the relationships between them; Level 5: ‘Deeper structural links’: recognition of whole pieces and some awareness of underlying structures; Level 6: ‘Mature artistic progression’ in which there is awareness of cultural and emotional context of musical composition (Ockelford et al., 2011, p. 178).

For the first author (NS), the experience of working with autistic young people in immersive, multi-sensory theater environments shaped her observations (Trimingham and Shaughnessy, 2016; Beadle-Brown et al., 2018). Shaughnessy had used the Sounds of Intent (SoI) Framework previously and refers to these categories in her field notes on the first workshop. She also uses terminology from the literature on masking and autism (Sedgwick et al., 2021; Pearson and Rose, 2023). The focus is on Jasmine (a 13-year-old autistic girl with selective mutism) who had previously participated in drama-based workshops. The sections in bold indicate where the commentary is particularly pertinent to the PP-Framework categorization (See Box 1).

As well as using some of the terms of the SoI framework (reactive, interactive), the language used to describe Jasmine’s creative engagement and behavior draws on autistic masking research, a topic that is attracting increasing interest, particularly in relation to autism and gender.

identity (Sedgwick et al., 2021; Pearson and Rose, 2023). This is important as a context for the PP-Framework. In its broadest terms, masking refers to performative strategies whereby an autistic individual adapts their behavior to appear ‘normal’ or neurotypical thereby concealing neurodivergent difference. There are a range of terminologies associated with masking (the overarching term preferred by the autistic community), including camouflage, compensation, adaptive morphing, passing and assimilation alongside conceptualizations that differentiate between these terms, particularly the tripartite social camouflage measure, the CATQ (Hull et al., 2017). This distinguishes between masking (concealing autistic features and performing a neurotypical persona); assimilation (blending in with others and trying not to be noticed as different) and compensation which involves forcing normative social behaviors (e.g., eye contact, shaking hands). Compensation is also used in the literature to describe the alternative strategies developed by some autistic people to ‘bypass socio-cognitive challenges’ (Livingston et al., 2019; Pearson and Rose, 2023). However, the description of Jasmine’s engagement refers to a range of performative behaviors and qualities of experience which fall outside of these paradigms: references to ‘furtive’, ‘playing to fit in’, ‘private’, not ‘feeling it’, and ‘actively listening.’ These observations (from drama and music informed perspectives) refer to an embodied understanding of participatory practice, attentive to the physicality of body language, gesture and facial expression. It was also evident that the sensory environment (high level of noise and cacophony) as well as group dynamics and relationships were impacting on Jasmine’s engagement and this situated perspective could not be divorced from the observations of her engagement. It was in this context that the PP-Framework began to emerge.

BOX 1 **Extract from field notes illustrating initial stage of development of PP-Framework**

*Jasmine (J): Alert, watching from side of room, observes others but **tries not to be noticed** (eyes averted, staying at the back of the room).*

*J is attracted by the box of stim toys, crossing the room to join group. Moves around the group (on outside) and is very interested in the contents of the box (**sensory engagement**); **seems at ease with group but prefers to be on the sidelines.** Chooses a multicolored squidgy ball. Returns to side of room to open the packet and finger the toy. A TA interacts, noting her interest.*

*J appears on camera seated **behind the keyboard, watching peers**. **Watching and listening to unstructured playing from others before group music; v passive**. Asked to move to put up “rules” sheet and obliges**. Very interested in percussion instruments, watching JW intently** as she offers each one to the group. J is offered choice of instruments by JW. Takes one and shows interest; **follows instructions on how to play; plays with instrument as object during unstructured setting up time. Reactive** as responds to JW*

*Another student requests her instrument and J relinquishes it (compliant). Then accepts drum being offered; **starts to tap the drum but in a way that is furtive as if she does not have permission and does not want anyone else to see**; it’s a **private activity**. She seems to be on **high alert in relation to the room** and peers but her **attention shifts when there is sound** (evident in eyes and body). Drum playing is **reactive**. She is doing what the others are doing, so trying to fit in and not to get noticed (**Assimilation**).*

*J has instrument under keyboard (stick); playing rhythm very quietly; but she is **watching and aware and interested** in JW (facilitator); **clearly engaged but not wanting to be seen**. She complies with the request to the group to stop playing; **does not appear to do the exercise but difficult to notice that she is not because of her masking**. Seems **v responsive to sound**; **the minute the playing starts again her engagement shifts; seems to be moving in time; embodied engagement with music (Interactive)***

*Name Song (turn taking). J is **responsive; she passes but raises her hand instead of name, cued by JW to “wave” and complies**.’ She **continues to listen, but not playing.** Her body language is very different when group music making stops; her **eyes follow sound for much of the time;** Attentive to JW introducing a copying a rhythm exercise: **watches JW demonstrate rhythm***

*A peer (Y) refers to J having a part in the ensemble and she nods assent to JW. Y gestures to J to bring her in: peer to peer interaction She is playing but seems to be **doing the action to fit in…tapping drum but not ‘feeling’ it, eyes down; hiding.** Listens to group discussion, planning the group composition; Y invites J to come in after her as they plan the group sequence.*

*J is becoming more engaged…**starts to play drum in time to accompany Y, feeling it, moving in time and attuned to group rhythm**; keenly and actively listening; stops with others. Complies with JW’s request: puts down drum as others pack away.*

In the video examples provided, blurred faces are required for anonymity purposes. However, in practice the filmed documentation featured facial expressions, bodily engagement and gesture, which are important to ensure the reliability of the interpretation. Taking the footage referred to in these initial observational notes, the following categories can be identified in Jasmine’s video clips (see Supplementary material-SF-for MP4 video clips):

In Video Clip 1 (See SF Video Clip 1 Listening, MP4), Jasmine moves from **‘not playing’** to a **‘reactive’** awareness of sound, transitioning to **interactive** engagement **and responsive play** as she starts to use a drumstick, tapping in time to the group rhythm. However, she does so privately and somewhat furtively. In Video Clip 2 **(Assimilation)**, we see Jasmine **‘playing to pass’**; she is beating with a stick but out of time with the group and appears to be doing so to blend in, to be seen to be playing in response to the facilitator’s instructions to beat a rhythm. In Video clip 3 (**Entrainment**), she is much more attuned to the group rhythm, so is **playing for pleasure in a group**, beating the drum and in time with the group, hence clearly engaged in contributing to the group music making.

### 3.2 Presentation of the framework and application in research context

As illustrated above, the PP-Framework emerged from the initial analysis of video data by researchers representing the multi-modalities of participatory arts. The PP-Framework developed from the ‘Playing A/Part’ research practices differs from SoI as it has arisen within a different context, capturing types of participation (interaction/non-interaction) in terms of performative behaviors and qualities of experience as modes of ‘playing’, rather than levels of musical skill/communication. The three behavioral domains from SoI (Reactive, Proactive, Interactive) are expanded in the PP-Framework to nine modes of play, in addition to a non-playing mode. Table 1 gives summary characteristics of each mode, mapping them against SoI. The PP-Framework delineates two Proactive modes and six Interactive modes. The “Reactive” Mode and Domain correspond across both models, and the first Mode, “Not Playing” is not mapped in SoI.^2^

**Table 1:** SoI domains mapped against modes of play in PP-Framework.

SoI domain	PP-Framework mode of play	PP-Framework example/explanation
	Not Playing	Deliberate disengagement; waiting for activity to begin; fiddling or engaging with materials that are not part of the workshop and its resources
Reactive	Reactive Play	Responding to sound and the sounds of another in a way that does not change the direction of the music. Consistent across PP and SoI frameworks. Examples include, shift in attention (evident in body movement, eye contact) or reaction to sound (hands over ears or putting on ear defenders).
Proactive	Autonomous Play	Making sound spontaneously in relation to the workshop materials and environment. A creative act: authorial and agential. For example, singing to self, spontaneously playing notes on a keyboard.
	Playing for Pleasure individually	Creative expression (via sound, movement) through sensorily driven (stims) or curiosity driven engagement. Authorial, agential with the individual absorbed and focused: in ‘flow’ (Csikszentmihalyi, 1990), i.e., immersive involvement. For example, repeatedly stroking the neck of a violin or twiddling with the cable of headphones, while appearing absorbed in the activity and unaware of presence of others; being immersed in exploring the different parts of an instrument.
	Playing for Pleasure individually with awareness of others’ presence	As above but showing clear awareness of presence of others.
Interactive	Playing for Pleasure in a group	Integrating sensorial, social and personal interest in the materials of the workshop environment—e.g., playing or singing along with others in a band while appearing absorbed and focused. Individual or group ‘flow’.
	Responsive Play	Responding with sounds to the sounds of another. May be authorial or imitative. For example: repeating the rhythm of another using body (hand or feet tapping), or their own instrument; interactive call-and-response techniques adding sounds to a texture such as a drumbeat, or riff, or melody.
	Playful Play	Playing to get a reaction. Musical heckles, teases which appear authorial and agential. Examples include: adding cheeky vocals alongside group instrumental playing; deliberate discordant or loud notes that disrupt or are added to a musical phrase.
	Transactive Play	Initiating play or creating changes to the direction of the creative activity. Authorial and agential. For example: playing a novel melody; offering a novel method of working (such as “conducting” the group); changing the tempo; adding a novel sound that changes the affect; suggesting another person starts the music (verbally enabling another), in all cases, motivating a response from others. Another example is free improvisation—simultaneously offering musical ideas while feeding back on others.
	Playing to Pass	Playing to comply: participating because they have been asked to do so alongside behavioral signs that they do not want to—socially driven, fitting in. Musical assimilation. For example, touching keys on a keyboard while other group members are also playing their instruments, but looking away or appearing disinterested or detached while doing this.

**Figure 1:**
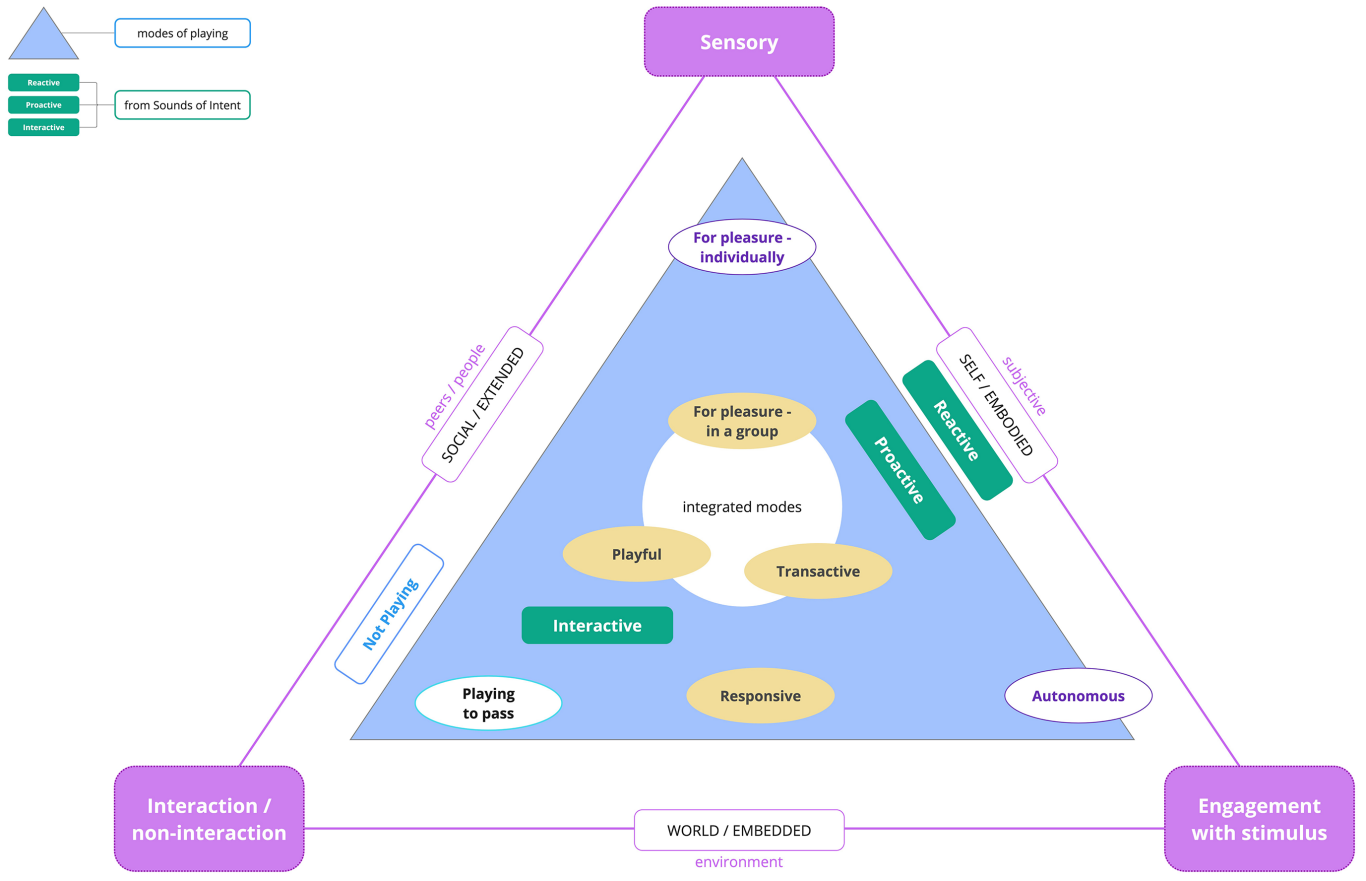
The PP-Framework.

Figure 1 constitutes a diagrammatic representation of the Framework showing where the modes of play are positioned, at or between the triangle apices. These represent privileged areas of focus within a social, sensory and creative space (“sensory,” “interaction/non-interaction” and “engagement with stimulus” on Figure 1). For example, ‘Playing for Pleasure (individually)’, in which attention is focused toward sensory experience is positioned at the top “sensory” apex, while Interactive play is situated between “Interactive-responsive/non-interaction” and “engagement with stimulus” apices, since it requires both social and musical engagement. The inner three modes of play are characterized by attentional focus which integrates the interactive, sensory and engagement with musical stimulus, although not to equal degrees. The outer triangle illustrates relational and ecological dimensions whereby activity is: (a) social, with peers and practitioners, and therefore extended beyond individuals; (b) situated in a social and creative environment, thereby embedded; and (c) is experienced subjectively via sounds, instruments and techniques, stimulating engagement that is embodied.^3^

### 3.3 Inter-rater reliability

**Figure 2:**
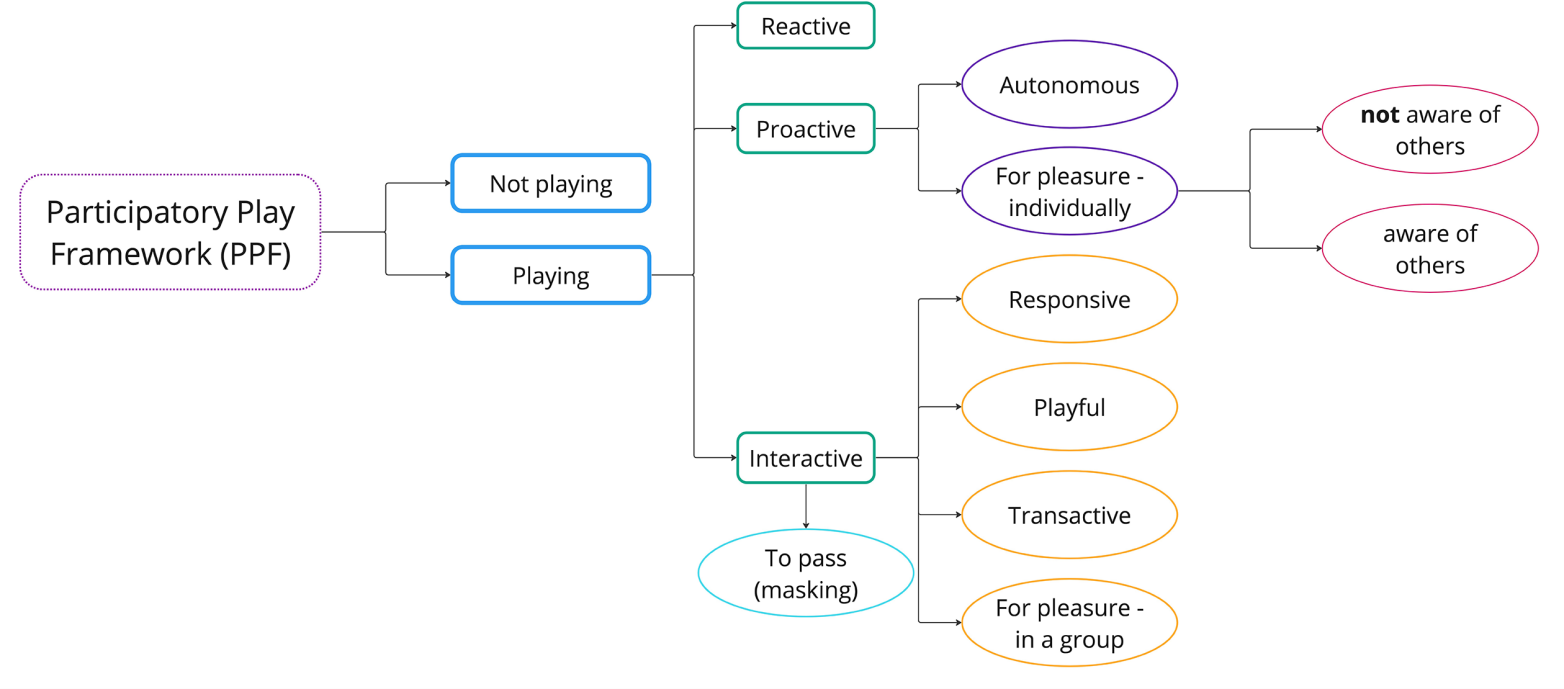
Decision tree for PP-Framework coding.

*Interdisciplinary discussion* in developing and refining the coding scheme as an interdisciplinary team (combining expertise in psychology, music, participatory arts, digital media and technology, autism and autistic lived experience), we met to watch and discuss a number of video clips that were rich in that they included several different play modes. The arts practitioners provided a commentary on what they were seeing in terms of different kinds of play, including the role played by the music itself in structuring and supporting engagement. In doing this, it became clear that the social scientists were unable to ‘read’ certain aspects of the context, in particular those relating to the affordance of the music itself in distinguishing between play modes. This highlighted the importance of having an arts practitioner with expertise in the relevant art form (in this case music) provide an initial coding of the videos to capture where this is essential to coding reliably (for example, imitation of rhythm, cases of improvisation, musical ‘teases’). These need to be noted in contextual information given to other coders without musical expertise. The social scientists highlighted where explicit behavioral criteria and contextual details (implicitly understood by the arts practitioners) were needed, in addition to those relating to music, to identify particular play modes. For example, interdisciplinary discussion identified the need for more detailed behavioral information to delineate “playing to pass” from “playing for pleasure in a group,” including behavioral evidence of disengagement (half-hearted/ mechanical actions and looking away appearing detached) while playing instruments in a group. The social scientists also suggested developing and using a decision tree relating to the levels of coding (1. playing versus not playing; 2. reactive, proactive, interactive; 3. sub-codes of proactive and interactive) to help scaffold and focus the coding (see Figure 2).

*Reliability coding* Interobserver agreement was assessed by having two members of the project team independently code the same 30.68 min of video footage in total, involving 6 different young people who had taken part in the music workshops. The video clips were selected to ensure the inclusion of sufficient examples of each play mode. One coder (an arts practitioner) had taken part in the music workshops and the other (from a social science background) had had no prior involvement in the music workshops. They were initially trained in the coding procedure using footage from 3 participants which covered 2 different workshops. Interobserver agreement coding was begun once a satisfactory overall percentage of 75% or more was reached during training between the coders and the first author (NS) who had coded all the footage used in training sessions. An overall Cohen’s kappa (Cohen, 1960) was computed to assess the level of agreement between the two raters in identifying the modes of play in the PP-Framework. This found substantial agreement, kappa = 0.78 (95% CI, 0.64 to 0.93, *p* < 0.001).

The following case study examples explore the different modes of playing, as represented by the PP-Framework.

#### 3.3.1 Encounters and engagement: shifting modalities within the first workshop

In the first example (see Video compilation 1), we see the progression of Maya (autistic 12-year-old) moving from **not playing** (stimming) through to composition, initially for her own pleasure and then in the context of the group. In the first excerpt, she is ‘**not playing**’ with the instruments, although she is not disengaged; she seems at ease in the environment, twiddling with a string (which is not part of the musical environment or equipment). This is appropriate to the stage of the session where the group are settling down and warming up as they adjust to the environment, each other and the creative context. The workshops took place after school as an optional activity for boarding school students (or for those doing an extended day). This meant they were situated in a liminal space between the structured school curriculum and down time in the evening (in boarding house or home). In the second excerpt, Maya is engaging by joining in with repeated notes and rhythm, hence shifting into **interactive responsive play** although she is compliant in responding to instructions and copying her peers. In excerpt 3 she is focused intently on copying the sequence of notes and the rhythm of a peer. She is actively joining in, rather than passively imitating as in the previous excerpt (2) and is very evidently seeking to play in a way that complements her peers (**interactive: playing for pleasure in a group**). In excerpt 4, we see Maya moving into a more **proactive** mode (**playing for pleasure individually with awareness of others presence**) as she becomes interested in making her own sequence of notes, improvising alongside others but absorbed in her own tune which emerges, over the others who stop playing to listen to what one of her peers describes as “beautiful.” In the final sequence, Maya is playing repeated chords above the group sequence in what can be described as **transactive play**, offering the group a form of chordal descant from a position of musical leadership that builds on her compositional breakthrough in the previous section (excerpt).

#### 3.3.2 Becoming an ensemble: from amoebas to cogs

Video analysis, and the music practitioner’s field notes were indicative of a trajectory in sociality, via group music-making, between Weeks 1 and 3. During these sessions, participants were introduced to the idea of creating beats as a group, playing rhythmic patterns that related through a common reference to an underlying pulse (interactive play). Repeatedly, the shared pulses set up at the start of the exercises rapidly disintegrated, resulting in every participant spinning into their own tempo. As the pulse collapsed, participants began to explore their instruments in more individually-focused ways; maintaining patterns but drifting from the shared pulse toward individual pulses or veering into exploratory play, sensory engagement with the instruments (e.g., running hands along the Ukelele strings, rubbing the drumhead). The effect was a blurring of musical pulse, which sounded confusing then chaotic. The disintegrating music with various pulses and evolving rhythms *could* have been overwhelming sensorially. It was tolerated through the coping strategies of individual focus and sensory exploration—which, in turn, *furthered* the sonic chaos (Video clip 5).

This occurred repeatedly for the first 2 weeks. In Session 3, there was a noticeable shift *in the music*, to a more stable pulse as participants became attuned rhythmically and a range of roles taken to produce a coherent but varied musical texture: a melodic riff created by the keyboard players was accompanied by a keyboard drone and unpitched percussion. These musically-differentiated responses to the task of creating a band Musical Signature enabled a sense of different voices contributing to a whole—the girls began to hear themselves as parts of a musical system (Video clip 5, 20s).

In Video clip 5, 20s, Bella, who had previously tied her ukulele rhythm to her own sense of pulse (**proactive-autonomous**), smiles to herself and nods her head in time to the rhythm as she plays (musically **interactive-responsive**, while the smile and rhythmically attuned, embodied play suggest **playing for pleasure in a group**). Eve (drum and keyboard) swapped instruments as we were about to practice the group composition. Instead of this act resulting in further exploration (spinning away from the group sound), Eve adopted the keyboard drone originally devised by Clare, who had stopped playing (Eve—**interactive-responsive**). Both Bella and Eve move toward increased group interaction in different ways.

While the enjoyment of the instrumental sounds was consistent with the pleasure of sonic explorations from the first weeks, at this point, the pleasure appeared to derive (in part) from a sound created as a band—a riff to which participants had contributed in varied ways (through distinct musical roles—drone, ukulele chords, percussion, keyboard melody), and which maintained its clarity through enhanced listening to one another and a shared objective.

The problem of the de-stabilized beat illustrates ways in which sensory preferences relating to the group’s musical sound come into play with a desire for sociality. An unclear beat is not easily tolerable, so the escape routes are toward private enjoyable experience (musical stimming/interest in the instruments or rhythms) or working *together* to create a stable pulse—an inherently interactive endeavor requiring awareness of the ensemble sound and musical attunement. Our coherent beat in week 3 was partly achieved through rhythmic entrainment (an ensemble skill which has developed), but driven by sensory preferences, for example, ending rhythmic chaos, and a desire for a social experience—being part of a band that makes coherent music.

**Figure 3:**
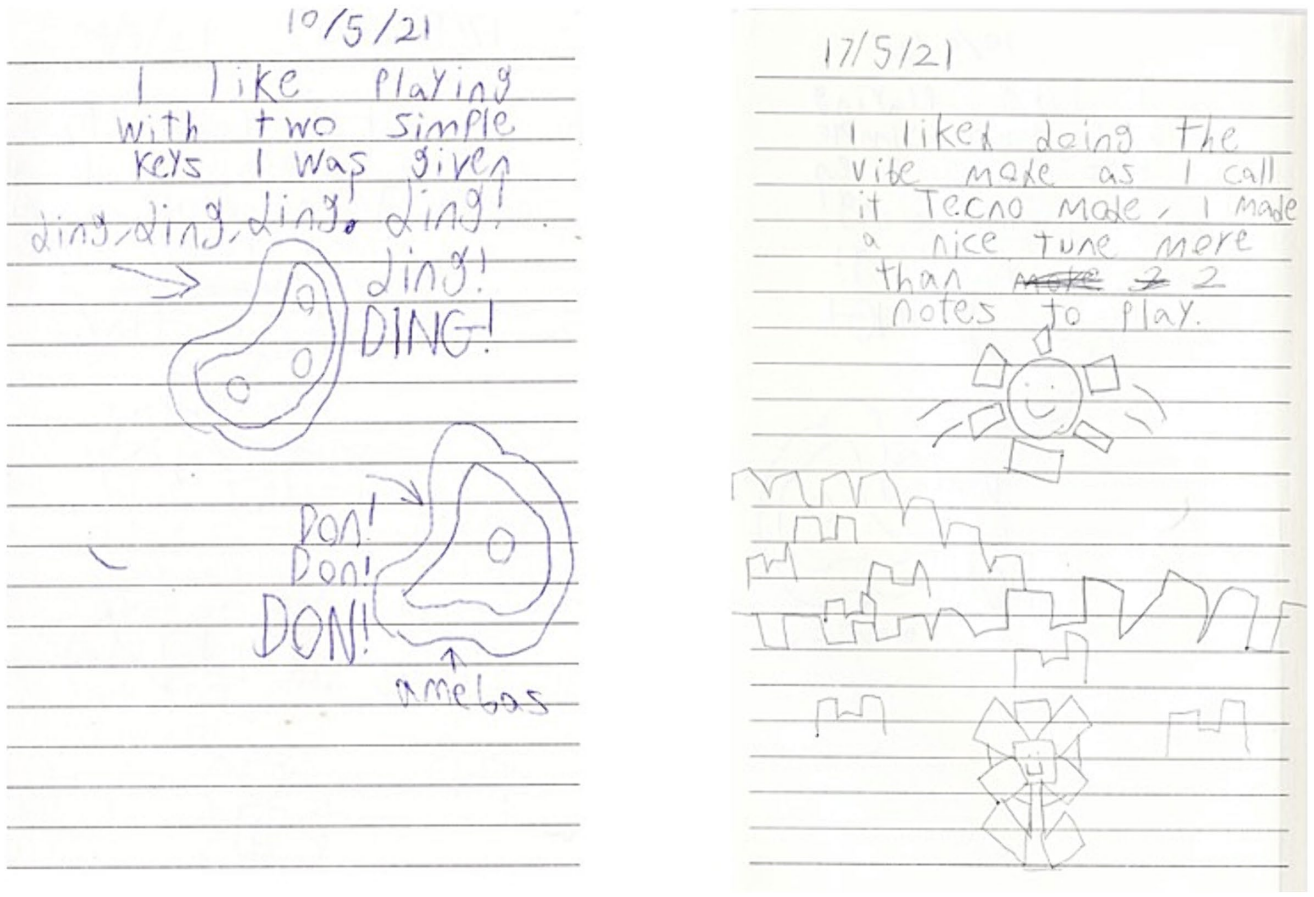
Participant diary extracts from weeks 1 and 2.

The amoebas and block landscapes in Figure 3^4^ are drawings from a participant’s project diary—amoebas after the first session, and the landscape a week later, perhaps reflecting a musical understanding that journeys from cellular (isolated notes) to systemic (ensemble textures).

While it is possible to trace a general group trajectory toward sociality through musical developments, the participatory volitional (conscious) or spontaneous (sub or preconscious) *strategies* of individuals are far more nuanced, operating in varied ways as the participants encounter the workshop activities. In Workshop 3 (Video clip 5, 20s), Bella takes pleasure in being part of a coherent ensemble beat (**playing for pleasure in a group**), Eve changes strategy from her recurrent exploratory (**proactive-autonomous**) and off-task music making (**playful**—see section below) to supporting the musical space with Clare’s drone (**interactive**). Sammy (keyboards, off camera) continues to play the main melody, occasionally contributing variations to it (**responsive**, being authorial in a group context), while Maya (keyboard—off camera) disengages during the group chatter and chaotic moments (**not playing**), but joins in, concentrating, when we begin playing (**interactive-responsive**)—perhaps enjoying being a part of the band (**playing for pleasure in a group**). Jasmine plays a keyboard, her output routed privately through headphones; she is unsure of the melody and does not wish to be heard (**playing to pass**). Clare (not on camera) does not join in this stage of the activity (**not playing**).

Routes of engagement toward the group composition were diverse and achieved through workshop-framed tasks as well as off-task music making. The PP-Framework encompasses all sounds created, whether through an engagement with a set task, an interest in sounds, sensory pleasure with stim toys or instruments, what is shared or not shared, provocations, authorship, meaning-making or interactive play. It enables an acceptance of all workshop sounds, which (following Gershon, 2011 and Feld, 1996) become an epistemological lens, through which connections may be forged, and routes to engagement understood.

#### 3.3.3 Negotiating public and private participatory stances and territories in ensemble musicking

Individual negotiation of private and public participatory stances and territories was apparent across ensemble music-making workshops, with a frequent, observable fluctuation between overt (public) creative contribution to group tasks and an intentional or spontaneous covert (private) retreat or detachment from them marked either by solo sonic/musical exploration [playing for pleasure (individual)] or a reduction in external awareness and increase in internal attentional focus—apparently ‘zoning out’ or daydreaming (not playing).

Eve (aged 12) had received ADHD and Autism Diagnoses. She was lively, articulate, a sophisticated mimic and possessed a strong sense of humor, frequently testing boundaries. In early workshops, she adopted two strategies to manage awareness of self and private/public boundaries. These comprised (a) playing or humming/singing quietly either in the gaps/breaks between group music-making episodes or (b) within textures while ensemble rehearsal was occurring. Both can be considered instances of assimilation that function to hide or limit evidence of musicality. Many of Eve’s early musical contributions were so surreptitiously effected that it was only after repeated viewings of the video data that this subtle layer of agency became visible. A clear distinction between private and public musical participation was apparent. Covert, ‘private’ off task contributions highlighted an enculturated musical response, in which aspects of musical syntax were clearly internalized (phrase structure, motifs) and stylistic awareness was evident in vocal improvisation and vocal inflections. Her practitioner-invited ‘public’ contributions to creative tasks were far more minimal and disengaged, such as taking on the role of providing a single drone note throughout an evolving piece (playing to pass). *Eve seemed to simultaneously want to be noticed and yet to hide.*

For example, in the third workshop she began by quietly inserting a piano melody she had been covertly exploring into a co-created ensemble composition (Video compilation 3, opening). The melody was in opposition to the tempo and key laid out by the rest of the group, suggesting a **playful mode** of play. However, when the ukulele player began a repeating chord sequence, she moved to improvisational quiet singing that perfectly complemented the harmonic structure (**interactive transactive play**) (09 s) but immediately stopped when this was noticed, looking suddenly abstracted (34 s). This alternation between private and public participatory stances continued throughout the session, with her tending to look at the facilitator during her covert contributions, checking if these were possibly being noticed (**playful play**). At the end of the workshop, the facilitator—aware of Eve’s partially detached response throughout the session—encouraged her to ‘be creative in a way that’s unique to you’ (2nd excerpt, at c.36-41 s). Eve initially gave a jokey verbal response, chanting ‘bang, bang, bang, bang’ while acting out playing the keyboard with one finger (42-46 s). However, she then began to play the keyboard melody she had inserted earlier (still extremely quietly), while the facilitator spoke to her—clearly offering a musical (unspoken) response to the facilitator’s observation. This was a significant turning point (46 s).

As the workshops progressed, this performing ‘in the gaps’ or covertly/privately became more overt, and integrated within ensemble tasks, marked by a raised volume level and greater eye contact (**interactive-playing for pleasure in a group**) (Video clip 6 at c.1.00-1.09) Eve increasingly chose to share her private musical identity, marked by a pull toward the group, and boundaries between her private and public music-making began to dissolve. This case study example illustrates the fluctuation between different modes of play and ways in which the balance between different modes may alter across a period of weeks, as the ensemble space becomes more familiar and perhaps easier to inhabit. The alternation between private and public participatory stances, sometimes interspersed with **not playing** or **playing to pass,** gradually incorporates increased instances of **playing to be playful, proactive playing for pleasure individually**, later becoming more **transactive** and overtly integrated into ensemble music-making.

### 3.4 Application of participatory play framework in a ‘real-world’ setting

The PP-Framework elucidates participation strategies which take account of creative practices and musical outputs in participatory arts workshops, and which may offer insights to practitioners when reflecting on the effectiveness of their work. Such reflection is frequently undertaken in the context of short evaluation sessions at the end of workshops. The following small scale exploratory study tested the PP-Framework as an evaluation tool for a team of musicians, including members of a professional chamber orchestra and classical conservatoire students, who delivered a creative music project. The workshop participants in this instance were adults living with the effects of homelessness attending a day-center in central London. Due to time constraints and the necessity of reporting to funders, arts-based project evaluation *can* become utilitarian in character,^5^ focusing on measuring outcomes against aims, (for example, improved engagement and/or wellbeing) alongside operational areas of success and what might be improved. In such questions, the detail of individuals’ *musical* engagement may become lost, yet interpreting nuances of musical engagement is precisely where the musicians’ expertise lies. We wanted to examine whether using the PP-Framework as a set of discussion prompts during post-workshop evaluations would capture musically astute observations and facilitate strategies of musical engagement, with the potential to contribute to staff development and improved practice.

#### 3.4.1 Procedures

The study objective was to explore the efficacy of the PP-Framework as a project evaluation tool for staff who gave informed consent to take part. The PP-Framework modes of play were used as focus group prompts during five staff debrief sessions, of 15–20 min’ duration, taking place immediately after each workshop. The 4th author (JW), who had contributed to the development of the PP-Framework for the ‘Playing A/Part’ project, led the focus groups and workshops. Focus group members were presented with the PP-Framework modes and descriptors (Table 1), which were explained verbally to them. The resulting discussions relating to their sense-making around workshop participants’ engagement through the ‘lens’ of the framework were recorded, transcribed verbatim and analyzed by authors 2 (RH; a music psychologist) and 4 (JW; participatory music specialist) using deductive thematic analysis (Braun and Clarke, 2006). Line-by-line coding at the semantic level was completed for each focus group transcript, the semantic codes identified were discussed iteratively by authors 2 and 4 and clustered according to similarity, resulting in three themes: (i) transition versus stasis of play modes; (ii) semantic widening of play modes, and (iii) potential of PP-Framework as a tool for self-reflection.

#### 3.4.2 Focus group membership and characteristics

The team of musicians consisted of orchestral musicians (*n* = 7), emergent workshop leaders (*n* = 2), and music students from a UK conservatoire (one undergraduate, one postgraduate, studying participatory music). This team composition was fluid, with individual musicians attending between one and five workshops and focus groups. Staff from the Day Center and orchestral management team attended all workshops and focus groups. Within each session, various degrees of familiarity with the project and PP-Framework were thereby present. Due to a limited timeframe in which to establish ethical practices with vulnerable adults and the fluid nature of music group membership/the drop-in structure of the sessions, the day center music group participants were informed of the study *via* a privacy notice prior to participating in the workshops, but not involved with the focus groups, which concentrated on staff perspectives. The participants who joined the music group (6 ≤ n ≤ 15 weekly) did so as *music makers*, and the music team did not hold knowledge of their medical or lived histories—observation and discussion remained grounded in how the participants engaged in the sessions. Ethical approval for this study was granted by the Royal Academy of Music Research Ethics Committee.

Focus group sessions were characterized by operational conditions that were typical of this and other similar projects:^6^ limited discussion time, a fluid multidisciplinary team, and reliance on recall of the workshop activity. There was, therefore, a risk of bias toward a focus on successful outcomes. For example, day center staff might have emphasized positive outcomes due to a desire for the project (funded by the orchestra and conservatoire) to continue. Nonetheless, the study trialed the PP-Framework as an evaluation tool situated in a participatory music project, with a team for whom the encounter with the PP-Framework was new. We present a few key illustrative extracts to support the themes identified.

#### 3.4.3 Findings: the PP-framework, its adoption and semantic shifts

Over the course of five focus groups (FG1-FG5), the musicians developed an increased confidence in their interpretation of the group activities using the PP-Framework modes of play to describe nuances of musical participation. Discussions shifted from descriptions of participants’ actions to an increase in citations of the PP-Framework modes, from four in FG1 to 10 in FG5. Phrases such as “Everyone in the circle was up for it” (describing **interactive-responsive** FG2) and “he had the agency [to play the drum] but he’d rather just listen” (**reactive**, FG2) both from musicians, refer to discrete moments of engagement. In the final session (FG5), a musician who had attended all sessions used the PP-Framework to observe shifts across different modes.

*Playing **for pleasure individually** and playing **to pass** – he did that back and forth a few times, but then it became playing **for pleasure in a group**; ... he wanted to get it right for everyone else. And there was a bit of noodling [**proactive-autonomous**], **playful** play where he’d get the tambourine, use it as a command to try to get everyone’s attention ... and also **not playing** [person left the room] – all in the space of 15 min!* (FG5. Charles, musician).

This suggests an increased confidence in the application of the PP-Framework over several sessions, toward an understanding of the fluidity of participation strategies: that the modes are attached to transitory behaviors, rather than to people. This enhanced comprehension could perhaps be accelerated through training for practitioners.

The musicians sometimes used the PP-Framework to refer to their own engagement:

*S (musician): I feel I would have interactive moments with the participants … it’s with the participants but also how we are feeling about out interactions as well.* FG2 Sarah.

The PP-Framework may function bi-directionally—as a self-evaluation tool (potentially for participants and the team) and as a way of reading the group’s interactions. Its affordance to trace changes in modes of play may function as a developmental tool for participants and practitioners—with approaches encompassing the authorial and agential, subjective, sensory and interactive as the gold standard.

In later focus groups the musicians referred on several occasions to participants “knowing what to do” or “getting it right,” identifying these as sources of pleasure, and linking them to the playing **for pleasure** individually/in a group and **playing to pass** (playing to comply/please):

*I wonder if there’s somewhere between playing **for pleasure** and playing **to please [pass]**? they get”” something that they know they can do and they enjoy it: part of that enjoyment is they are not going to get it wrong [playing to pass]... I think Marcus [participant] ... wants to get it right, he really enjoys that, but part of the enjoyment that he gets is knowing that other people can work with him.* (FG3. Charles, musician).

This represents a semantic widening of playing **for pleasure,** in which the musicians comingle social, sensory and intellectual pleasures—all of which are plausible experiences in creative musicking. For Marcus (participant), the pleasure in getting it right for others is socially-derived. Another participant, Mira, repeated a three-note melody for the first two sessions without ever changing it (in an improvisation context). She appeared to have *mastered* the melody on her instrument, smiling as she played. Her pleasure may encompass the intellectual pleasure of learning. Both forms of pleasure involve attentional focus on the social environment (working with others) and the workshop materials (a three-note riff) rather than the attentional focus on *sensory* pleasure originally captured by playing **for pleasure** in the study with autistic girls, and significant to the lived experiences of neurodivergent people. For the musicians, “pleasure” is interpreted more broadly.

#### 3.4.4 The PP-framework as a post-workshop evaluation tool

The PP-Framework appears to facilitate an evaluative discussion based upon musicians’ ability to interpret and articulate musical experience. As the team became increasingly familiar with the PP-Framework, they began to customize it, entailing semantic widening. Such semantic differences are considered as follows: Over five sessions, returning members of the team used the PP-Framework modes with increased confidence, interrogating some of the mode boundaries and suggesting further variations within them. Such customization may indicate the PP-Framework’s robustness and utility as an evaluation tool that draws upon musical expertise. However, semantic widening of the **playing for pleasure** modes to include intellectual pleasures of mastery or social pleasures of musical sharing/being reliable in a team loses the centrality of sensory experience to those with sensory sensitivities or diversities of sensory processing. **Playing for sensory pleasure** may be a clearer descriptor. Additionally, widening the meaning of **playing to pass** to include getting it right (mastering the rhythm—pleasing the person who taught it to you) misses the connection to the originally conceived notion of **playing to pass** as a camouflaging strategy—playing to *comply*, to appear to fit in, associated with masking in autistic social experience. While customization of the framework may reflect its utility, semantic drift could be prevented by undertaking such customization with cognizance of the original project intentions.

## 4 Discussion

### 4.1 Summary of key findings

Analysis of the video-data collected from participatory music workshops conducted with autistic girls led to music and theater specialists in dialog with autism experts becoming attuned to different modes of performativity being played out through the music-making process. This culminated in the development of an innovative PP-Framework, which provides useful information for researchers using creative practices as an investigative tool by distinguishing different modes of performative behavior. These situated behaviors are important to identify as they have implications for data collection and reliability, as well as enriching research insights. Application of the PP-Framework to recorded video data from the music workshops (with female autistic adolescents) enhanced our understanding of social camouflage strategies and sensory experience for this population by enabling us to distinguish between different modes of playing (e.g., Playing to Pass, Playing for Pleasure individually or in a group) alongside existing categories associated with established empirical frameworks (e.g., the Sounds of Intent tripartite structure of reactive, interactive, proactive domains).

The PP-Framework was also capable of capturing changes in engagement, for example increases in authorial agency, self-expression and attunement to other people via group music-making, across a series of workshops. It also helped elucidate convergence and divergence in routes to change taken by different participants. This has the potential to inform us further about mechanisms of change in PAB approaches, in addition to providing evidence of that change. The framework was able to be adapted for use in a ‘real-world’ (not research) context; shifting the evaluation discourse toward a detailed appreciation of participants’ engagement that drew on the musicians’ expertise and lived experiences, including strategies that were on-and off-task. However, use of the PP-Framework in this ‘real-world’ context ascertained a need for further work to refine the modes of play and ensure reliability in their application. The ‘not playing’ category, for example, encompassed a range of dis/engagements. We decided that stimming with an object not brought to the workshop was ‘not playing’ in contrast to stimming with an object or instrument that was part of the workshop environment or resources which could be coded as ‘playing for pleasure.’ The rationale here was the play we were coding was musical play or any play related to this. However, if ‘not playing’ was to be more finely coded then we would be able to code, for example, sensory play unrelated to the workshop materials and combine it with ‘playing for pleasure individually’ if we were particularly interested in looking at how much sensory play as a whole was engaged in.

### 4.2 Evaluating participatory practices: engagement, creativity and change

In evaluating PAB projects, a series of debates and tensions have arisen between the disciplinary positions of arts and health science in relation to fundamental differences in their contexts and values (including esthetic and ethical values), as well as the kinds of beneficial changes sought. The status of the work as art involves judgments of artistic merit and skill which may run counter to the socially engaged or therapeutic context in which it was produced. In this project, we were interested in attributes of different art forms as well as bringing about positive change. This was articulated in our aims to ‘explore creativities’ and to ‘develop musical skills’ as a means of supporting ‘artistic agency’ and ‘creative empowerment’ through music as a vehicle for self-expression. As an evaluation tool, the PP-Framework is sensitive to creative, performative and behavioral aspects of PAB approaches, as well as to the kinds of changes sought through this work. It also draws on the strengths of artists and practitioners to offer nuanced and detailed accounts of practice, recognizing the complexities of their and others involvement, as well as the artistic process and the situational context in which the arts activities take place. Underpinning our research was an overarching aim to investigate the identities and experiences of autistic girls through creative practices. In addition to being a valuable tool for evaluation, the PP-Framework functioned as a tool of discovery, helping us find out more about the subjective lived experiences of the participating autistic girls, which in turn has the potential to inform the development of care and educational structures so that they are more attuned to the sensitivities of this group.

There are strong synergies between participatory community models and arts-based approaches in terms of values and objectives, but we recognized and embraced the challenge of data assembly as an arts/science collaboration. Practice-based approaches are acknowledged as democratizing and inclusive as they enable diverse modes of expression and communication, moving beyond the verbal modalities that dominate traditional qualitative data. ‘Artful data’ (Ellingson and Sotirin, 2020: 88) can be embodied, visual, material, immersive, offering richness and nuance through its articulation and representation of lived experience. This, however, also brings challenges of interpretation and reliability.

### 4.3 Relationship of findings to existing research

The PP-Framework was initially informed by the Sounds of Intent (SoI) model designed to map musical development in special needs contexts. SoI is, however, differently inflected to our PP-Framework which was created in a different context to identify and distinguish between participatory modes of play-based and performative engagement. The PP-Framework is also likely to be more widely applicable to other PAB approaches beyond music. SoI puts musical behavior at the heart of observations. The behaviors described relate to musical ontologies (e.g., “imitates the sounds made by someone else,” “produces musical motifs”), so that the model tracks development through music-making: music is valued as an activity in and of itself, and not only as a route to wellbeing or improved cognition or communication. There are no descriptions of talking behaviors, or measures of the acquisition of verbal/ written language, for example. Our work in ‘Playing A/Part’ aligns with this epistemological approach in the sense that sounds and musical behaviors form part of the project data. However, while SoI was a valuable analytical model, we found that further differentiations between types of Proactive and Interactive strategies were required to understand the lived experience of autistic girls and to capture their styles of engagement, which acknowledged the positive-and negatively-valenced effects of the musical-sensory-social workshop environment.

Although we did not aim to assess participants’ musical development (as compared to the six developmental Levels in SoI), our project *was* broadly developmental for those taking part.

Progression entailed an increased confidence and authorial agency, attunement to others, a developed interest in music, as well as an awareness of different sounds and how they might be put together. This was evidenced both in videographic analysis captured by the PP-Framework and the development of individual compositions which were installed in “sound-dens” at the end of the project. SoI may be applied to any genre of music making. However, the framework is grounded in Zygonic theory, and progression through the levels depends on a developing perception of *relationships between sounds*, in particular, apprehending patterns and motifs., During the music workshops that explored creating the sounds of a spooky forest using found objects, audio effects and a looper, girls created soundscapes by layering, juxtaposing and contrasting sounds, inviting a creativity driven by narrative, drama, and an atmospheric evocation of imaginary place. Within Zygonic theory, this is a type of creativity that constitutes an area for further development.

### 4.4 Limitations, recommendations and next steps

The testing of the PP-Framework in a real-world setting suggested that more is needed to ensure that the modes of play descriptors can be reliably used. In doing reliability testing there were differences between the researchers with experience of creative practice (who concurred) and those without who needed more contextual information to code reliably. This suggests there is a need to develop training materials that support awareness of communicative cues that are non-verbal, gestural, embodied and that convey participants engagement and attentional focus. Additionally, such training would encompass awareness of what to look for in the environment and the relational context. Further development is required to test the PP-Framework in other contexts and to adapt it for other arts practices, particularly the performative modalities of drama and movement. There are some aspects of the process that require particular consideration in terms of reliability. It is important for an expert in the art form to be the first to code the documentation. The reliability of this could be separately established by getting more than one arts expert to code the material. This can then be provided to non-experts as part of the context to inform their coding of the play modes.

While we experienced difficulties in implementing standardized measures in this project, we do not mean to imply that these cannot be used in relation to PAB projects. Rather, we suggest that more work is needed into how they can be used more effectively, particularly with young people, given the very different contexts and values characteristic of PAB approaches. Arts, science, and youth perspectives need to be brought into reciprocal dialog to examine how barriers might be reduced and/or overcome to improve their implementation and efficacy. Going forward, arts-science collaborations might usefully work on the development of measures better able to pick up the kinds of changes which take place in PAB projects, as well as how to best capture long-term change.

## 5 Conclusion

The PP-Framework evolved through an awareness of different modes of engagement in workshop space and how these are indicative of autistic characteristics (observed/reported in project), particularly those associated with girls where under-diagnosis has been attributed (in part) to social camouflage strategies (masking, compensation, assimilation). The use of arts-based practices to investigate autistic girls’ identities and experiences offered rich insights into the nuances of social creativity and performativity as aspects of autistic behavior. The researchers addressed the challenge of how to recognize, document and analyze these modes of ‘playing’ in ways that acknowledge both artistry and identify autistic features so that the data could be used as discovery, to consider what this tells us about autistic experience and creativity. The PP-Framework is a transdisciplinary model, developed through collaboration between researchers in social science and arts, combining evidence-based practice and practice-based evidence. The research provoked reflection on researcher roles and the tensions between co-construction, imposition and power structures. The PP-Framework has the potential to be a two-way facing tool, with application for both practitioners and participants. It affords a defined theoretical space for considering modes of agency and interaction. This is the first iteration of what is an evolving framework, working toward the development of a protocol for using measures and tools.

## Data availability statement

The data for this study are included in the article/supplementary material. Additional data, supporting the study are available from https://data.kent.ac.uk/id/eprint/506. Some data may not be publicly available due to ethical constraints given the sensitivity of working with vulnerable groups. Further inquiries can be directed to the corresponding author/s.

## Ethics statement

The studies involving humans were approved by University of Kent Central Research Ethics Committee. The studies were conducted in accordance with the local legislation and institutional requirements. Written informed consent for participation in this study was provided by the participants’ legal guardians/next of kin and informed assent was provided by the participants.

## Author contributions

NS: Conceptualization, Formal analysis, Funding acquisition, Investigation, Methodology, Project administration, Resources, Validation, Writing – original draft, Writing – review & editing. RH: Conceptualization, Formal analysis, Investigation, Methodology, Project administration, Resources, Writing – original draft, Writing – review & editing. EW: Conceptualization, Formal analysis, Funding acquisition, Investigation, Methodology, Project administration, Resources, Supervision, Validation, Writing – original draft, Writing – review & editing. JW: Conceptualization, Formal analysis, Investigation, Methodology, Project administration, Resources, Validation, Visualization, Writing – original draft, Writing – review & editing. RJ: Conceptualization, Funding acquisition, Investigation, Methodology, Project administration, Validation, Visualization, Data curation, Writing – review & editing. HN: Conceptualization, Investigation, Methodology, Project administration, Validation, Data curation, Writing – review & editing.

## Footnotes

^1^For a discussion of findings emerging from analysis of journals and semi-structured interviews, see Herbert, R. [forthcoming] Sonic Lives. In Herbert, R., Shaughnessy, N., and Williams, E. (eds) *Beyond Autistic Stereotypes: New Perspectives on Identies, Gender and Experience.* Oxford: OUP.

^2^No reaction to sound is possible in SoI at level 1, if no auditory processing takes place in the participant.

^3^As the Participatory Play Framework and associated resources are undergoing further refinement and testing, they can be accessed via the Kent Data Repository (KDR) upon request. The URI link is https://data.kent.ac.uk/id/eprint/506. The Parts/OM measures are included in Supplementary Materials.

^4^Figure 3 was originally published in Walduck (2024), and has been reproduced under Creative Commons BY 4.0 with permission from The British Journal of Music Education, Cambridge University Press.

^5^Solutions to evaluation narrowness are being addressed increasingly throughout the sector, for example, through the work of the Center for Cultural Value based at the University of Leeds in the United Kingdom. https://www.culturalvalue.org.uk. While many evaluation models laudably propose co-creation of evaluation design with a range of stakeholders, few evaluate with respect to the intricacies of human experience encoded within the artistic/musical outcomes of such projects.

^6^The orchestra and workshop leader have worked with people attending several different day centers for those living with the effects of homelessness for 25 years. Further details may be found online: https://www.asmf.org/learning-posts/making
