# Supplementary material for: Playing with data differently: engaging with autism and gender through participatory arts/music and a performative framework for analysis
Source: Front Psychol. 2024 Jun 17;15:1324036. doi: 10.3389/fpsyg.2024.1324036 (PMC11218808; doi:10.3389/fpsyg.2024.1324036)
Supplement: Supplementary file 1 [file Table_1.DOCX]

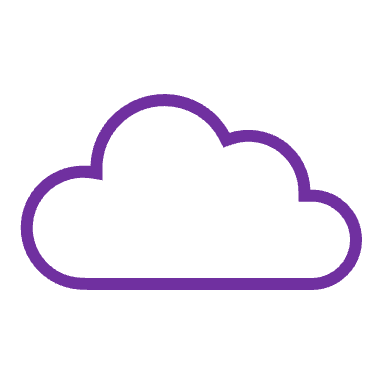

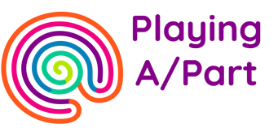


**Vibe Check**

**Vibe Check**

| Name: | Date: |
| --- | --- |
| Session: | Time in workshop:  **Start** |

**Instructions:**

Below are a number of statements with which you may agree or disagree.

Beneath each statement is a five point scale of emojis (Please see key below).

Please circle the emoji closest to what you think or feel right now. Only circle **ONE** emoji for each statement.


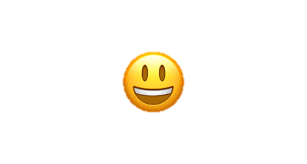

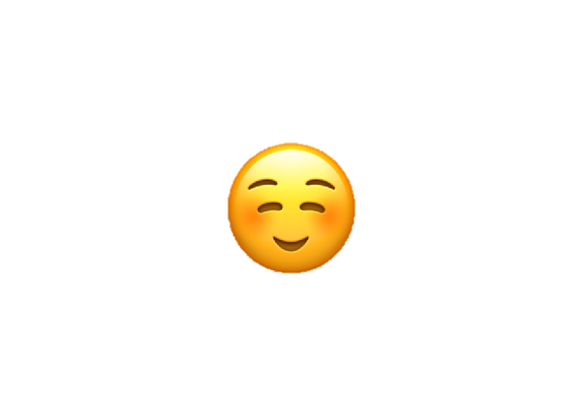


| 1 | 2 | 3 | 4 | 5 |
| --- | --- | --- | --- | --- |
| Not at all true for me | Not true for me | Sort of true for me | True for me | Very true for me |


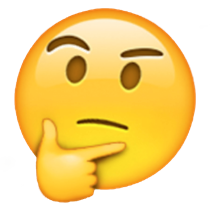

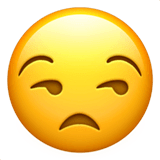

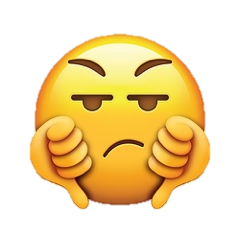


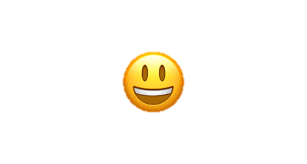


1. **Right now I am feeling confident in myself**


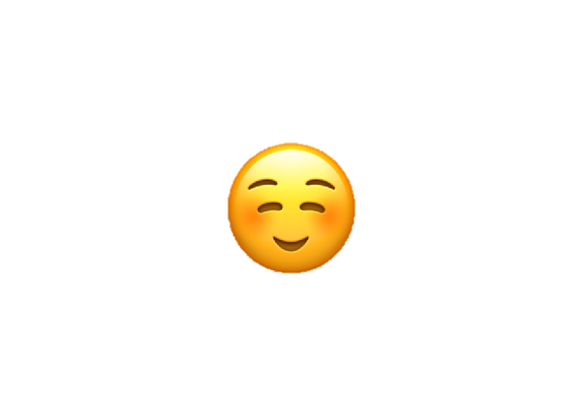

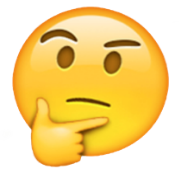

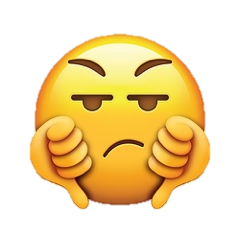

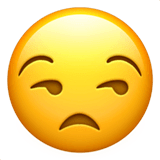


| 1 | 2 | 3 | 4 | 5 |
| --- | --- | --- | --- | --- |
| Not at all true for me | Not true for me | Sort of true for me | True for me | Very true for me |


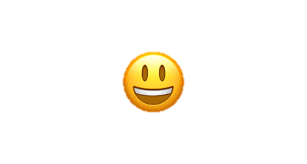

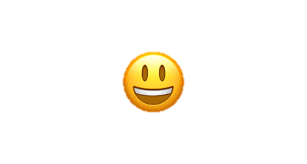

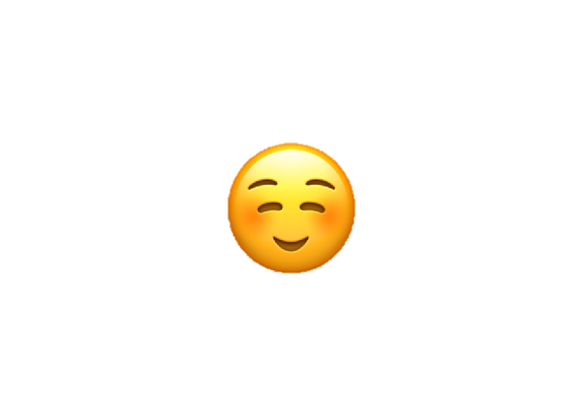

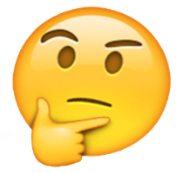

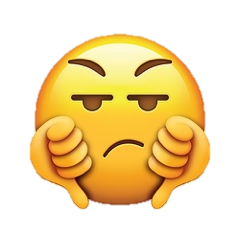

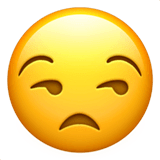

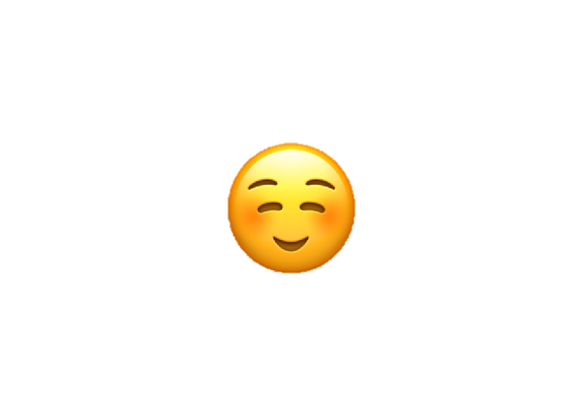

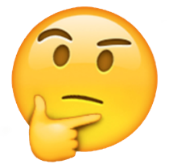

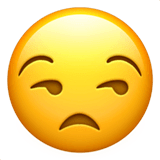

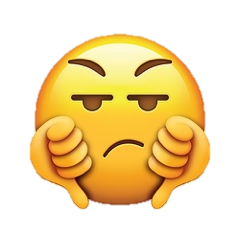


1. **Right now I feel I can speak up**

| 1 | 2 | 3 | 4 | 5 |
| --- | --- | --- | --- | --- |
| Not at all true for me | Not true for me | Sort of true for me | True for me | Very true for me |

| 1 | 2 | 3 | 4 | 5 |
| --- | --- | --- | --- | --- |
| Not at all true for me | Not true for me | Sort of true for me | True for me | Very true for me |

**3. Right now I feel I can be myself (I don’t have to pretend to be someone I am not)**

**
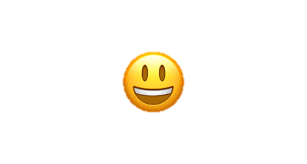

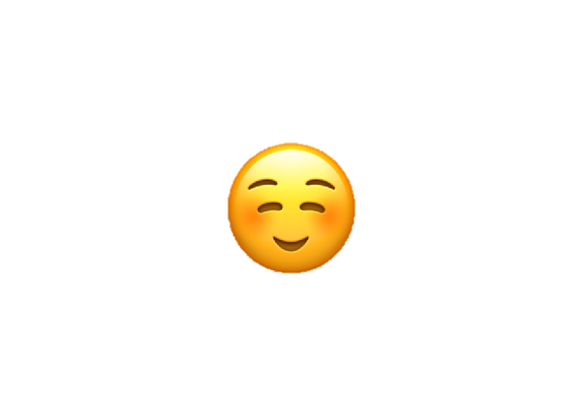
**
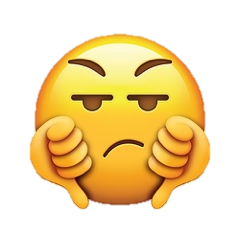
**4. Right now I am finding it easy to get on with the other students in the zoom workshop**

Right now I feel I can be myself


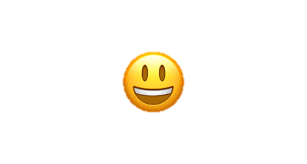

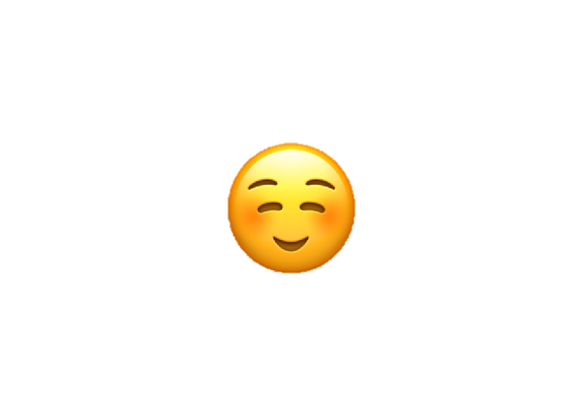

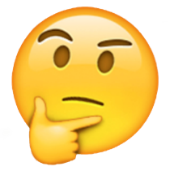

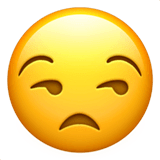

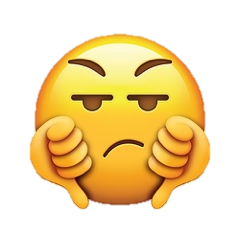

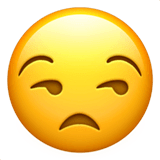

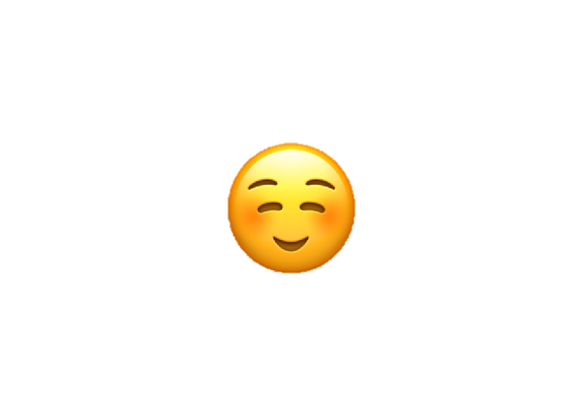

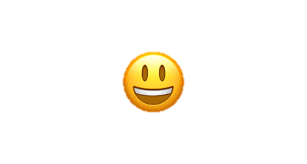

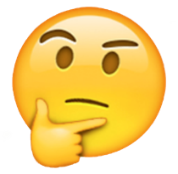

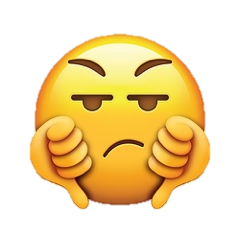

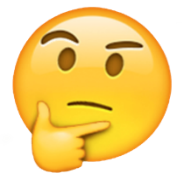

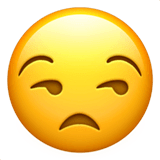


1. **Right now I am feeling creative**

| 1 | 2 | 3 | 4 | 5 |
| --- | --- | --- | --- | --- |
| Not at all true for me | Not true for me | Sort of true for me | True for me | Very true for me |

| 1 | 2 | 3 | 4 | 5 |
| --- | --- | --- | --- | --- |
| Not at all true for me | Not true for me | Sort of true for me | True for me | Very true for me |

| 1 | 2 | 3 | 4 | 5 |
| --- | --- | --- | --- | --- |
| Not at all true for me | Not true for me | Sort of true for me | True for me | Very true for me |

1. **Right now I am finding it easy to talk to the other students in the zoom workshop**
