# Supplementary material for: Playing with data differently: engaging with autism and gender through participatory arts/music and a performative framework for analysis
Source: Front Psychol. 2024 Jun 17;15:1324036. doi: 10.3389/fpsyg.2024.1324036 (PMC11218808; doi:10.3389/fpsyg.2024.1324036)
Supplement: Supplementary file 2 [file Table_2.DOCX]

**PArts/OM**

Participatory Arts Outcome Measure

**Practitioner version**

| Name of student:  Name of Practitioner: | Date:  Workshop: |
| --- | --- |

The PArts/OM aims to assess perceived **changes in self-confidence, self-expression, agency, peer social engagement, and/or creativity** in autistic girls/adolescents that have been brought about by their taking part in participatory arts’ workshops.

Below are a number of statements in relation to each student under consideration with which you may agree or disagree. Beneath each statement is a seven-point scale (**strongly disagree;** **disagree; somewhat disagree; neither agree nor disagree; slightly agree; agree; strongly agree**). Please indicate your level of agreement with each item by circling (if using paper version) or highlighting (if completing online) the appropriate response.

1. **Levels of self-confidence**

During the session **as a whole** the student appeared self-confident.

| Strongly disagree | Disagree | Somewhat disagree | Neither agree nor disagree | Slightly agree | Agree | Strongly agree |
| --- | --- | --- | --- | --- | --- | --- |

During the session the student’s confidence level appeared to improve.

| Strongly disagree  [Very clear no signs of change] | Disagree | Somewhat disagree | | Neither agree nor disagree | Slightly agree | Agree | Strongly agree  [Very clear signs of change] |
| --- | --- | --- | --- | --- | --- | --- | --- |
|  |  | | Please provide details [optional] | | | | |

1. **Levels of self-expression**

During the session **as a whole** the student appeared to find it easy to express themselves.

| Strongly disagree | Disagree | Somewhat disagree | Neither agree nor disagree | Slightly agree | Agree | Strongly agree |
| --- | --- | --- | --- | --- | --- | --- |

During the session the student’s willingness to express themselves appeared to increase.

| Strongly disagree  [Very clear no signs of change] | Disagree | Somewhat disagree | | Neither agree nor disagree | Slightly agree | Agree | Strongly agree  [Very clear signs of change] |
| --- | --- | --- | --- | --- | --- | --- | --- |
|  |  | | Please provide details [optional] | | | | |

1. **Levels of initiation**

During the session **as a whole** the student appeared to find it easy to start conversations/suggest activities or ideas.

| Strongly disagree | Disagree | Somewhat disagree | Neither agree nor disagree | Slightly agree |  | Agree | Strongly agree |
| --- | --- | --- | --- | --- | --- | --- | --- |

During the session the student’s willingness to start conversations/suggest activities or ideas appeared to improve.

| Strongly disagree | Disagree | Somewhat disagree | | Neither agree nor disagree | Slightly agree | Agree | Strongly agree |
| --- | --- | --- | --- | --- | --- | --- | --- |
|  |  | | Please provide details [optional] | | | | |

1. **Levels of peer social engagement**

During the session **as a whole** the student appeared to find it easy to work with/talk to other students in the group.

| Strongly disagree | Disagree | Somewhat disagree | Neither agree nor disagree | Slightly agree | Agree | Strongly agree |
| --- | --- | --- | --- | --- | --- | --- |

During the session the student’s willingness to work with/talk to other students in the group appeared to improve.

| Strongly disagree | Disagree | Somewhat disagree | Neither agree nor disagree | Slightly agree | Agree | Strongly agree |
| --- | --- | --- | --- | --- | --- | --- |
| Please provide details [optional].  Include information here [required] on whether the girl was working in a group with other students at school or in a room of their own (whether at home or school) | | | | | | |

1. **Levels of creativity**

During the session **as a whole** the student appeared to be engaging with the workshop tasks in a creatively meaningful way.

| Strongly disagree | Disagree | Somewhat disagree | Neither agree nor disagree | Slightly agree | Agree | Strongly agree |
| --- | --- | --- | --- | --- | --- | --- |

During the session the student’s willingness to engage with the workshop tasks in a creatively meaningful way appeared to improve.

| Strongly disagree | Disagree | Somewhat disagree | | Neither agree nor disagree | Slightly agree | Agree | Strongly agree |
| --- | --- | --- | --- | --- | --- | --- | --- |
|  |  | | Please provide details [optional] | | | | |

| **OPTIONAL**  **Please add any observations/ comments below on anything else you perceived to have changed through the girl’s participation in the workshops not covered by the items above.**  **Please also comment on any changes you would make to this outcome measure.** |
| --- |

| **OPTIONAL**  **Please add any observations/ comments below on anything that emerged from the workshop process that was insightful in terms of identity, experience, sensory preferences and creative interests?** |
| --- |
